# The roles of METTL3 on autophagy and proliferation of vascular smooth muscle cells are mediated by mTOR rather than by CDK1

**DOI:** 10.1186/s13008-023-00096-5

**Published:** 2023-08-09

**Authors:** Hanshen Luo, Xingliang Wu, Bo Huo, Liyuan Liu, Ding-Sheng Jiang, Xin Yi

**Affiliations:** 1 Division of Cardiothoracic and Vascular Surgery, Tongji Hospital, Tongji Medical College, Huazhong University of Science and Technology, 1095 Jiefang Ave., Wuhan, 430030 Hubei China; 2 Department of Cardiology, Renmin Hospital of Wuhan University, Jiefang Road 238, Wuhan, China; 3 Key Laboratory of Organ Transplantation, Ministry of Education, NHC Key Laboratory of Organ Transplantation; Key Laboratory of Organ Transplantation, Chinese Academy of Medical Sciences, Wuhan, Hubei China

**Keywords:** Vascular smooth muscle cell, Autophagy, Proliferation, METTL3, mTOR, CDK1/CDC2

## Abstract

**Background:**

Aberrant proliferation of vascular smooth muscle cells (VSMCs) is the cause of neointima formation followed by vascular injury. Autophagy is involved in this pathological process, but its function is controversial. Recently, we found that methyltransferase like 3 (METTL3) inhibited VSMC proliferation by activating autophagosome formation. Moreover, we also demonstrated that METTL3 reduced the levels of phosphorylated mammalian target of rapamycin (p-mTOR) and cyclin dependent kinase 1 (p-CDK1/CDC2), which were critical for autophagy and proliferation regulation. However, whether mTOR and CDK1 mediated the function of METTL3 on autophagy and proliferation in VSMCs remains unknown.

**Results:**

We showed that the activator of mTOR, MHY1485 largely reversed the effects of METTL3 overexpression on VSMC autophagy and proliferation. Rapamycin, the inhibitor of mTOR, obviously nullified the pro-proliferation effects of METTL3 knockdown by activating autophagy in VSMCs. Unexpectedly, mTOR did not contribute to the impacts of METTL3 on migration and phenotypic switching of VSMCs. On the other hand, by knockdown of CDK1 in VSMC with METTL3 deficiency, we demonstrated that CDK1 was involved in METTL3-regulated proliferation of VSMCs, but this effect was not mediated by autophagy.

**Conclusions:**

We concluded that mTOR but not CDK1 mediated the role of METTL3 on VSMC proliferation and autophagy.

**Supplementary Information:**

The online version contains supplementary material available at 10.1186/s13008-023-00096-5.

## Background

Vascular smooth muscle cell (VSMC) proliferation is necessary for maintaining the structure of blood vascular system. However, both over-proliferation and insufficient proliferation of VSMCs have adverse effects on the body. For example, over-proliferation of VSMCs will lead to neointima formation, while insufficient proliferation will result in vascular structure disorder or even rupture. Autophagy was reported to be involved in VSMC proliferation and survival [1, 2]. However, the effect of autophagy on VSMC proliferation is controversial. It is well known that autophagy is a multiple steps biological process, the accumulation in autophagosomes may be due to increased autophagosome formation or impairment of autophagic flux degradation [3–5]. Excessive autophagosomes and impaired autophagic flux may have detrimental impacts on cells, or even results in autophagic cell death [6, 7], while benign autophagy activation is beneficial for cell survival and proliferation [8].

Our recently findings demonstrated that methyltransferase 3 (METTL3), a major N6-adenosine-methyltransferase, inhibits the proliferation, migration and synthetic phenotype of VSMCs by positively regulating autophagosome formation [9]. Whether autophagy is suppressed during cell mitosis is remains controversial [7, 10, 11]. For example, we previous results demonstrated that enhancer of zeste homolog 2 (EZH2) and euchromatic histone lysine methyltransferase 2 (EHMT2) promoted VSMC growth by inhibiting autophagosome formation [7, 10]; however, JIB-04, a pan-inhibitor of the Jumonji demethylase superfamily, suppressed VSMC proliferation by impairing autophagic flux [11]. Recently, Odle et al*.* revealed that both autophagy initiation and mammalian target of rapamycin complex 1 (mTORC1) were repressed during mitosis of cancer cells, and cyclin dependent kinase 1 (CDK1, also known as CDC2) substituted for inhibited mTORC1 as the master regulator of autophagy during mitosis [12]. Interestingly, we found that phosphorylation of both CDK1 and mTOR were downregulated by METTL3 overexpression in VSMCs [9]. Both CDK1 and mTOR are not only the key regulators of proliferation, but also critical for autophagy [11, 13]. However, whether CDK1 and mTOR mediated the function of METTL3 on VSMC proliferation and autophagy remains unknown.

In the present study, we found that mTOR activation largely abrogated the inhibitory effects of METTL3 overexpression on proliferation by suppressing autophagosome formation. On the contrary, mTOR inhibition nullified the pro-proliferation effects of METTL3 knockdown in VSMCs. However, neither mTOR activation nor inhibition has impact on the function of METTL3 on migration and phenotype switching of VSMCs. In addition, although METTL3 enhanced the phosphorylation of CDK1, CDK1 did not mediate the role of METTL3 on autophagy.

## Results

### mTOR mediated the effects of METTL3 on VSMC proliferation and autophagy

Recently, we reported that METTL3 inhibited the proliferation, migration, and synthetic phenotype of VSMCs by activating autophagosome formation [9]. We also found that METTL3 overexpression obviously inhibited, while METTL3 deficiency enhanced the phosphorylation of mTOR [9]. Given that mTOR is a well-known autophagy regulator but beyond this [14], we curious about whether mTOR functions as the target of METTL3 to mediate its function. Thus, to verify whether mTOR inactivation is required for the role of METTL3 on autophagy regulation and VSMC differentiation, the mTOR activator MHY1485, and mTOR inhibitor rapamycin were used to treat HASMCs with or without METTL3 overexpression or knockdown.

**Fig. 1:**
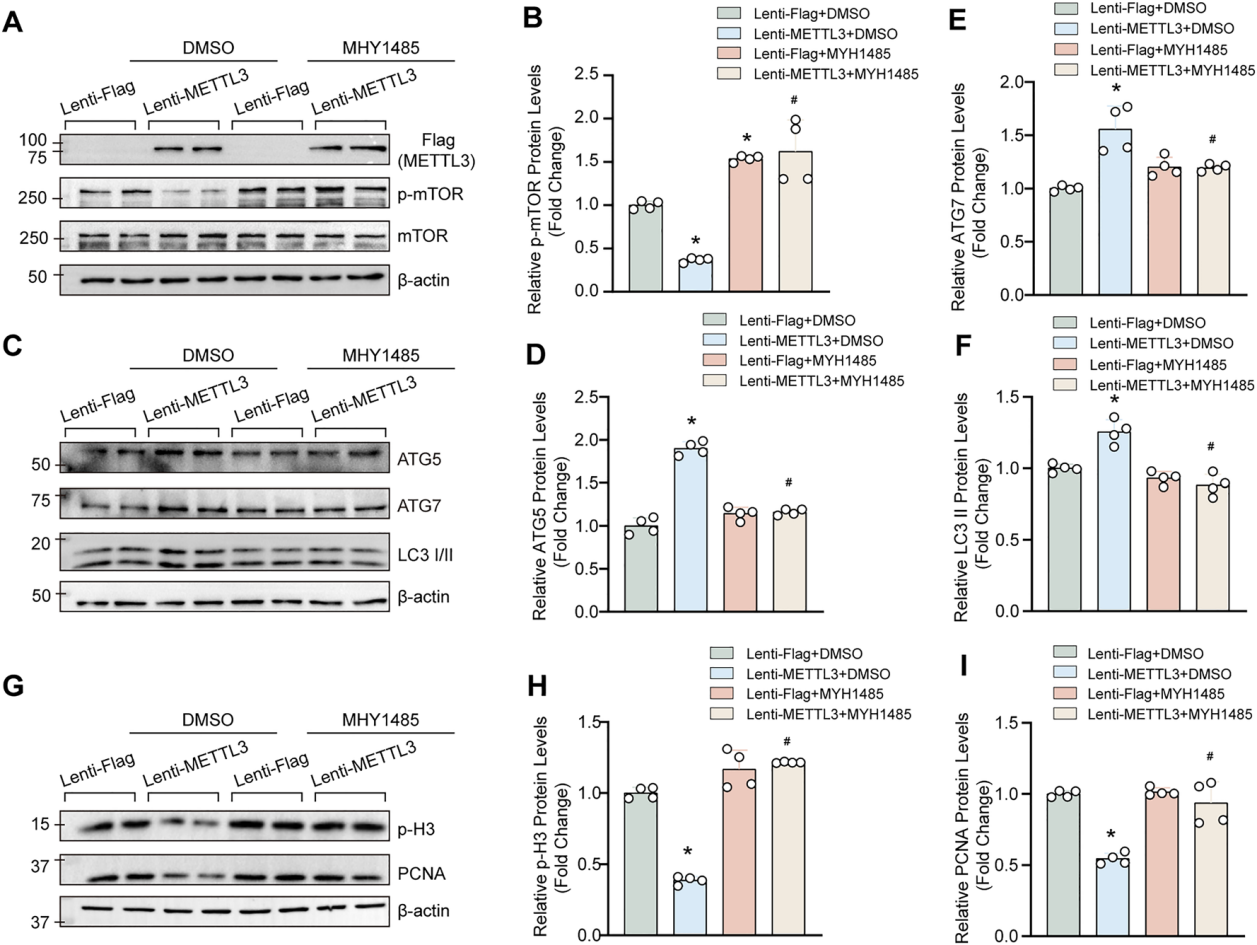
Treatment with mTOR activator MHY1485 counteracted the effects of METTL3 on autophagy and proliferation of VSMCs. **A** and **B** The expression levels of METTL3, p-mTOR, and mTOR in HASMCs with or without METTL3 overexpression and MHY1485 (10 µM) were examined by using western blotting (A); Quantitative results (**B**) (n = 4). **C**-**F** The protein levels of autophagy markers ATG5, ATG7, and LC3 were detected by using western blotting in the HASMCs with indicated treatments (**C**); Quantitative results of blots in C (**D**–**F**) (n = 4). **G**–**I** The representative blots of proliferation markers p-H3 and PCNA were detected in HASMCs with indicated stimulations (**G**); Quantitative results (**H** and **I**) (n = 4). β-actin serves as loading control. *p < 0.05 versus lenti-Flag + DMSO. #p < 0.05 versus lenti-METTL3 + DMSO

Our results showed that in DMSO treated HASMCs, METTL3 overexpression noticeably inhibited the phosphorylation of mTOR, and MHY1485 significantly increased the level of p-mTOR in HASMCs with or without METTL3 overexpression (Fig. 1A and B). Then, the mRNA levels of ATG5 and ATG7 were upregulated in HASMCs with METTL3 overexpression (Additional file 1: Figure S1A and B). MHY1485 had no effects on autophagy under normal condition, but MHY1485 downregulated the protein levels of ATG5, ATG7 and LC3II that enhanced by METTL3 overexpression, which indicated that MHY1485 negatively regulated METTL3-dependent autophagy (Fig. 1C–F). Similarly, compared with DMSO, MHY1485 had little effect on the expression of proliferation markers PCNA and p-H3 (Fig. 1G–I). Interestingly, MHY1485 treatment largely reversed the inhibitory effects of METTL3 overexpression on PCNA and p-H3 levels (Fig. 1G–I). Therefore, these results indicated that MHY1485 counteracted the impacts of METTL3 on autophagy and proliferation of VSMCs by activating mTOR.

**Fig. 2:**
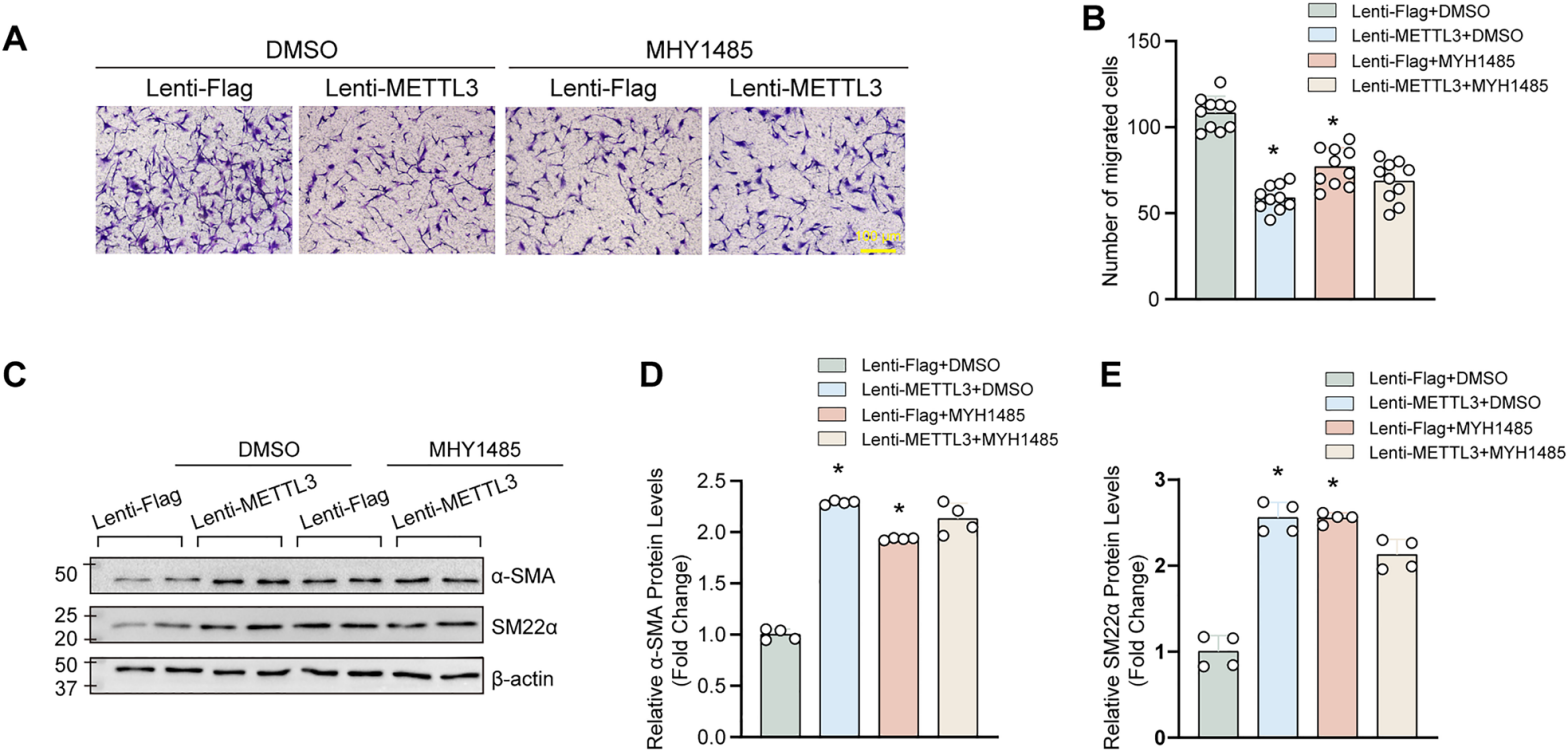
MHY1485 negatively regulated migration and synthetic phenotype of VSMCs, but cannot nullify the effects of METTL3 overexpression on these biological processes. **A** and **B** The HASMCs with lenti-METTL3 or lenti-Flag infection were treated with DMSO or MHY1485 (10 µM) for 24 h, and the migration of HASMCs was measured by using transwell assay (**A**); The quantitative results of migrated cells per high power field (**B**) (n = 10). Scale bar: 100 μm. **C**–**E** The protein levels of contractile markers α-SMA and SM22α were evaluated by western blotting (**C**); (**D** and **E**) The quantitative results of blots in C (n = 4). β-actin serves as loading control. *p < 0.05 versus lenti-Flag + DMSO

Except for proliferation and autophagy, METTL3 also suppressed migration and phenotypic switching of VSMCs. To investigate whether mTOR involved in these functions of METTL3, MHY1485 was used to treat HASMCs with or without METTL3 overexpression. Surprisingly, the results in Fig. 2A and B showed that MHY1485 treatment decelerated the migration of HASMCs with lenti-Flag. On the other hand, HASMCs treated with MHY1485 predisposed to be contractile phenotype, as evidenced by increased α-SMA and SM22α expression (Fig. 2C–E). These results suggested that similar to METTL3 overexpression, MHY1485 negatively regulated migration and synthetic phenotype of VSMCs. Furthermore, MHY1485 treatment cannot counteract the effects of METTL3 overexpression on migration and contractile phenotype, and no synergistic effects was found (Fig. 2).

**Fig. 3:**
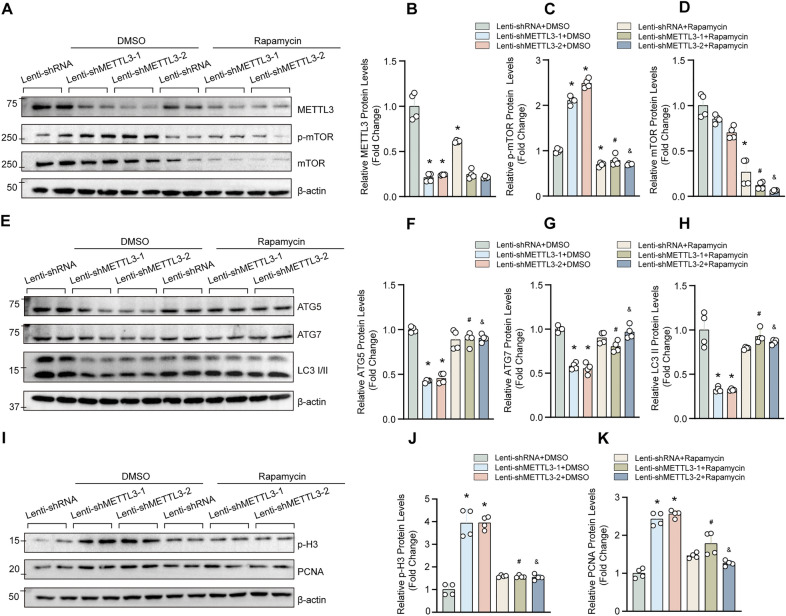
Rapamycin offset the impacts of METTL3 knockdown on autophagy and proliferation. **A**–**D** The representative blots of METTL3, p-mTOR and mTOR in HASMCs with indicated treatments (rapamycin: 150 nM); **B**–**D** Quantification analysis of METTL3, p-mTOR and mTOR protein levels in A (n = 4). **E**–**H** The protein levels of ATG5, ATG7 and LC3 were detected by using western blotting in HASMCs with indicated stimulus (**E**); **F**–**H** Quantitative results of E (n = 4). **I**–**K** Western blotting was used to detect the protein levels of proliferation markers p-H3 and PCNA in HASMCs with indicated treatments (**I**); **J** and **K** Quantitative results of blots in I (n = 4). β-actin serves as loading control. *p < 0.05 versus lenti-shRNA + DMSO; #p < 0.05 versus lenti-shRNA + Rapamycin; &p < 0.05 versus lenti-shRNA + lenti-shMETTL3-2

**Fig. 4:**
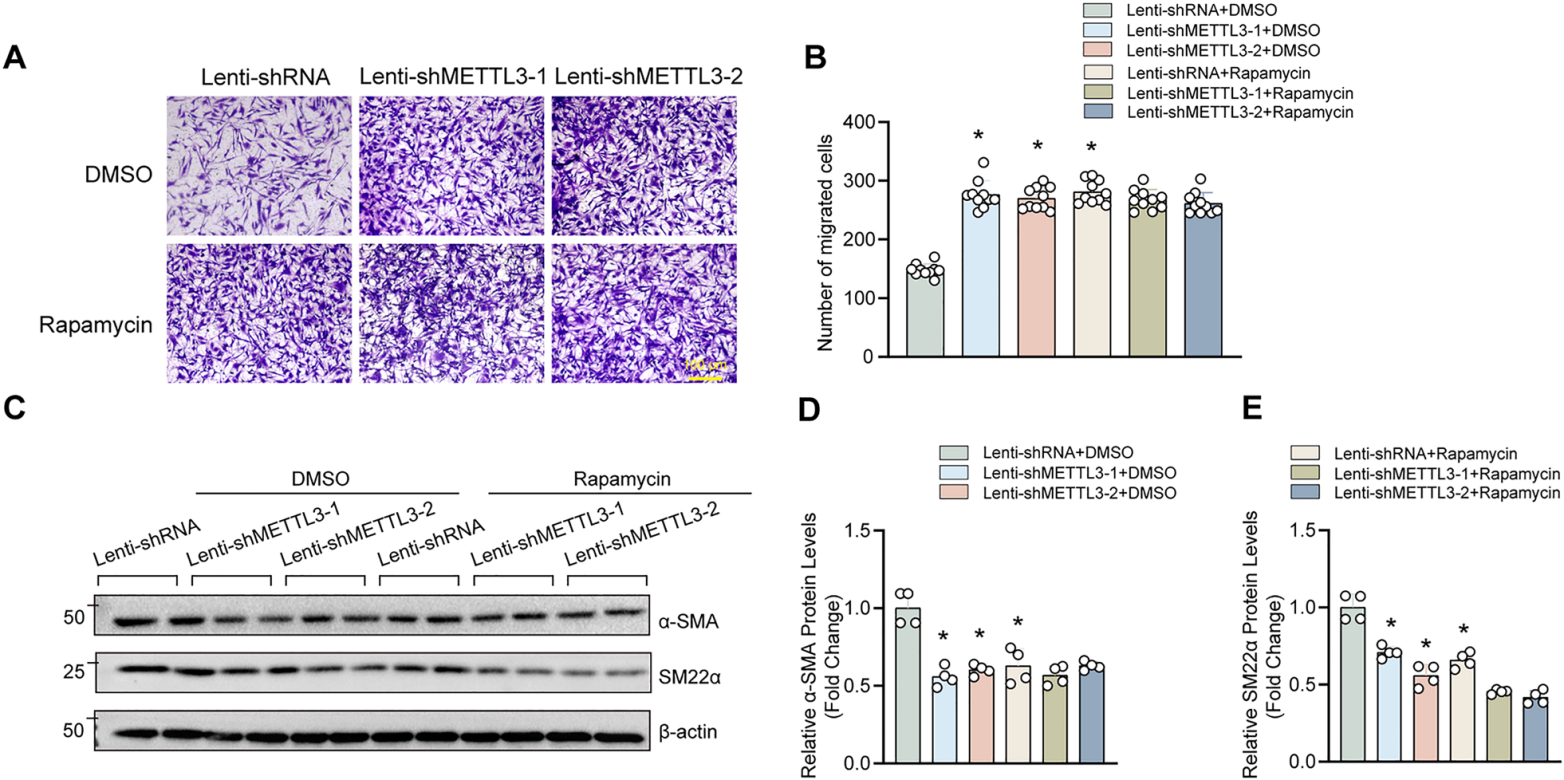
Rapamycin promoted HASMC migration but did not abrogate the effects of METTL3 on HASMC migration and synthetic phenotype. **A**–**B** Transwell assay were used to assess the migratory capacity of HASMCs treated with DMSO or rapamycin (150 nM) for 24 h after METTL3 knockdown or not, and the cells were stained with crystal violet (n = 10). Scale bar: 100 μm. **C**–**E** The protein levels of contractile markers α-SMA and SM22α were evaluated by western blotting (**C**); **D** and **E** Quantitative results of blots in C (n = 4). β-actin serves as loading control. *p < 0.05 versus lenti-shRNA + DMSO

Similarly, compared with METTL3 knocked down HASMCs treated with DMSO, mTOR inhibitor rapamycin largely offset the impacts of METTL3 knockdown on autophagy and proliferation, as evidenced by the reversed expression of ATG5, ATG7, LC3II, p-H3 and PCNA in HASMCs with METTL3 deficiency (Fig. 3A–K). In addition, although rapamycin can promote the migration of HASMCs, it did not abrogate the effects of METTL3 on HASMC migration and synthetic phenotype (Fig. 4A–E), which was consistent with the conclusion obtained from MHY1485. Therefore, these results indicated that mTOR may require for the function of METTL3 on autophagy and proliferation, but not necessary for migration and synthetic phenotype regulation.

### CDK1 is not required for METTL3-dependent autophagy regulation

**Fig. 5:**
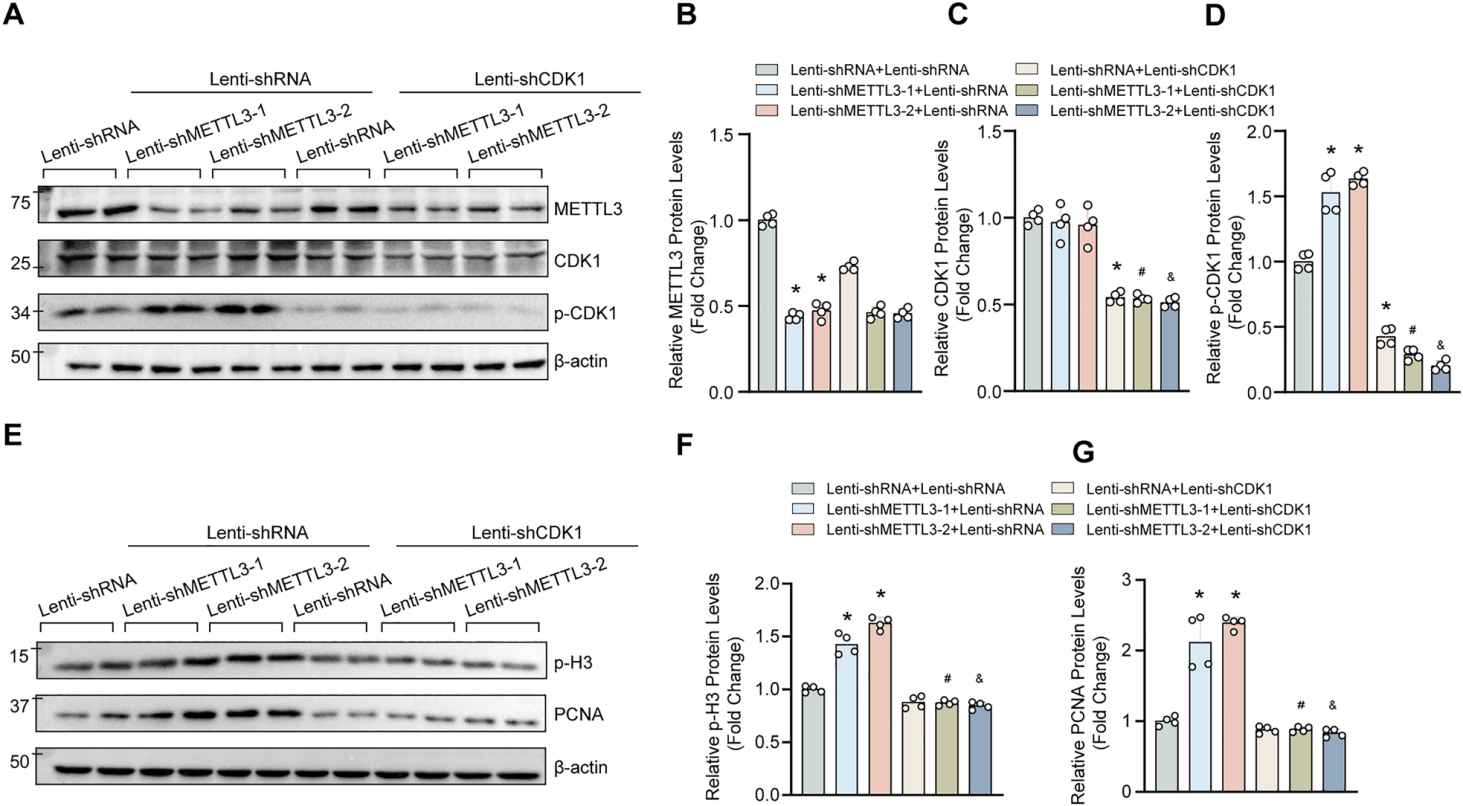
CDK1 knockdown eliminated the pro-proliferative effect of METTL3 on VSMC. **A**–**D** The protein levels of METTL3, CDK1 and phosphorylated CDK1 (p-CDK1) were evaluated by using western blotting in HASMCs with METTL3 and CDK1 knockdown or not (A); (B-D) Quantitative results of blots in A (n = 4). **E**–**G** The expression levels of proliferation markers p-H3 and PCNA were measured in HASMCs with indicated treatments by using western blotting (**E**); **F** and **G** Quantitative results of indicated blots in E (n = 4). β-actin serves as loading control. *p < 0.05 versus lenti-shRNA + lenti-shRNA; #p < 0.05 versus lenti-shRNA + lenti-shMETTL3-1; &p < 0.05 versus lenti-shRNA + lenti-shMETTL3-2

**Fig. 6:**
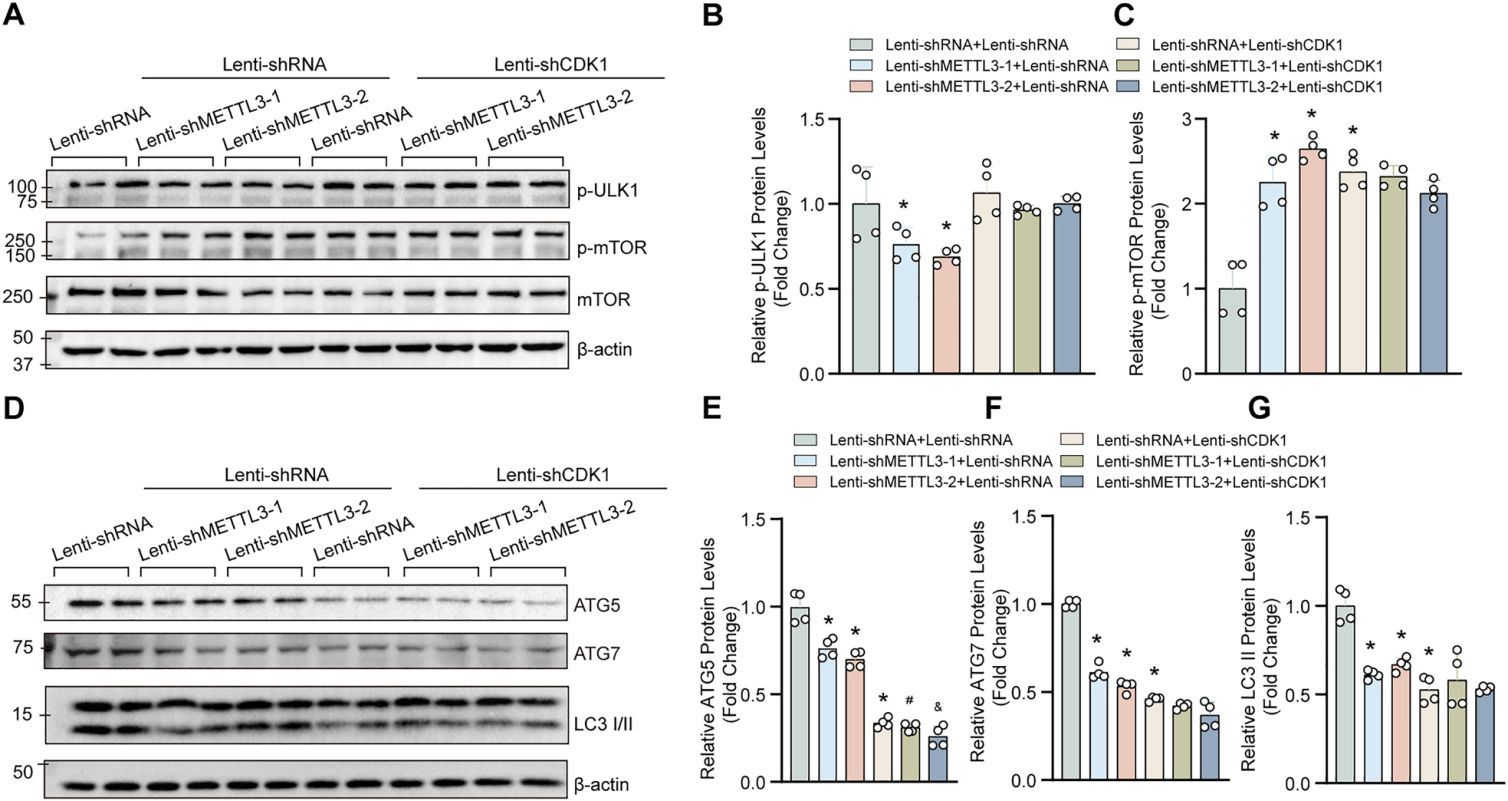
CDK1 involved in the regulation of autophagy but did not reverse the function of METTL3 on autophagy in VSMC. **A**–**C** Representative western blots and quantification results of phosphorylated ULK1 (p-ULK1) and p-mTOR (n = 4). **D**–**G** The expression levels of ATG5, ATG7 and LC3 in HASMCs with the indicated treatments (**D**); **E**–**G** Quantitative results of indicated blots in D (n = 4). β-actin serves as loading control. *p < 0.05 versus lenti-shRNA + lenti-shRNA; #p < 0.05 versus lenti-shRNA + lenti-shMETTL3-1; &p < 0.05 versus lenti-shRNA + lenti-shMETTL3-2

In addition to mTOR, our previous results also demonstrated that METTL3 overexpression inhibited, but METTL3 deficiency promoted CDK1 phosphorylation [9]. CDK1 has been recognized as a key cell cycle regulator during mitosis, and CDK1-cyclin B1 complex controls the cell cycle G2-M transition [15]. Recently, several studies have revealed that CDK1-induced the phosphorylation of ULK1 promotes mitotic autophagy and cell cycle progression [16]. Thus, to verify whether CDK1 mediated the role of METTL3 on proliferation and autophagy in VSMCs, we knockdown of CDK1 in HASMCs with or without METTL3 deficiency (Fig. 5A–D). Furtherly, we concluded that CDK1 was significantly knockdown at the protein and mRNA level (Additional file 1: Figure S2A–C). Our results showed that CDK1 knockdown largely eliminated the pro-proliferation effects of METTL3 knockdown on VSMCs (Fig. 5E–G). Intriguingly, our results revealed that compared with lenti-shRNA, CDK1 knockdown significantly enhanced the protein level of p-mTOR, while had little impact on p-ULK1 (Fig. 6A–C). Moreover, the expression levels of LC3II, ATG5 and ATG7 were decreased in HASMCs with CDK1 knockdown (Fig. 6D–G), suggesting that CDK1 may inhibit autophagy in VSMCs by activating mTOR. Unexpectedly, CDK1 is involved in the regulation of autophagy, but CDK1 knockdown cannot reverse the function of METTL3 on autophagy in VSMCs (Fig. 6D–G). Therefore, our results demonstrated that CDK1 is essential for the role of METTL3 regulating proliferation, but not for METTL3-dependent autophagy regulation.

## Discussion

**Fig. 7:**
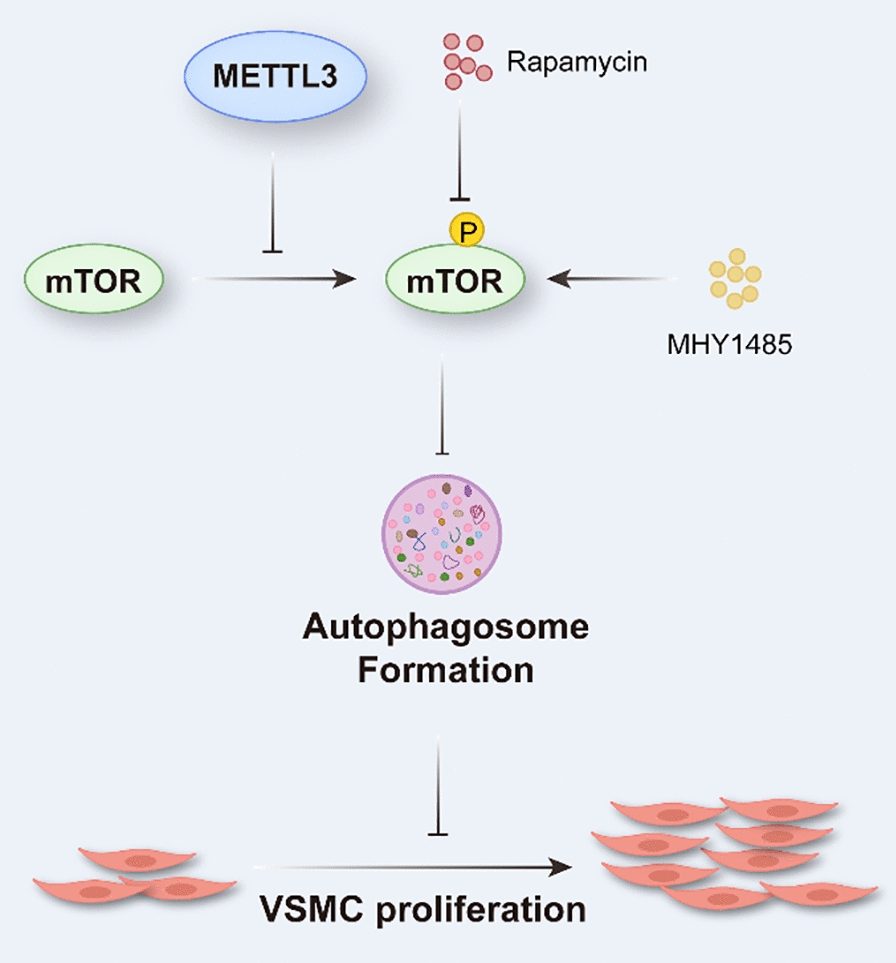
The indispensable role of mTOR in METTL3 regulation of autophagy and proliferation in VSMC. In VSMC, the inhibitory effect of METTL3 on proliferation by enhancing autophagosome formation is mediated by mTOR activation. The mTOR inhibitor, rapamycin, offsets the impacts of METTL3 knockdown on autophagy and proliferation. Similarly, MHY1485 counteracts the effects of METTL3 on VSMC autophagy and proliferation by activating mTOR

Herein, we reported that METTL3 noticeably inhibited the phosphorylation of mTOR and CDK1. mTOR activation reversed the impacts of METTL3 overexpression, and mTOR inhibition nullified the effects of METTL3 knockdown on autophagy and proliferation of VSMCs, but not migration and phenotypic switching. However, CDK1 knockdown can offset the pro-proliferation effects of METTL3 deficiency, but had no obvious impact on METTL3-dependent autophagy. Thus, our results suggested that the inhibitory effect of METTL3 on proliferation by enhancing autophagosome formation is dependent on mTOR activation (Fig. 7).

N6-Methyladenosine (m^6^A) RNA methylation is found widely across species and participated in abundant of biological processes regulation, such as proliferation, apoptosis, pyroptosis and ferroptosis [17–20]. RNA m^6^A was also found to involved in the VSMC differentiation, phenotypic switching, proliferation, and migration [21–23]. METTL3 is a primary methyltransferase that catalyzes m^6^A RNA methylation [24]. We demonstrated that METTL3 inhibited VSMC proliferation, migration, and phenotypic switching [9]. However, the role of METTL3 in cell proliferation is controversial. Although METTL3 promotes the proliferation of most tumor cells [25], there are always exceptions. For example, Deng et al*.* revealed that METTL3 suppressed colorectal cancer cell proliferation and migration by negatively regulating p38/ERK pathways [26]. METTL3 reduced cell proliferation through inducing cell cycle arrest at G0/G1 phase to attenuate proliferative vitreoretinopathy [27]. This controversy also exists in the field of METTL3-regulated heart regeneration. Gong et al. showed that loss of METTL3 promoted cardiomyocytes cell cycle re-entered, infract size decreased and cardiac function improved after myocardial infarction by reducing primary miR-143 m^6^A modification [28]. Similarly, Jiang et al. revealed that Mettl3 deficiency obviously increased cardiomyocyte proliferation and facilitated heart regeneration following heart injury in neonatal and adult mice [29]. On the contrary, Zhao et al*.* demonstrated that METTL3 pretreatment accelerated cardiomyocyte proliferation and reduced cardiomyocyte apoptosis under hypoxic or myocardial infarction conditions [30]. A possible reason for this phenomenon is that RNA m^6^A is widespread at over 25% of human transcripts, and different stimuli may cause m^6^A modification in different RNAs or different sites, leading to different outcomes.

mTOR is a protein kinase that involved in regulating cell growth, survival, metabolism, autophagy, and immunity [31]. Activation of mTOR promotes cell growth, and its inhibitors have been developed to treat cancer and cardiovascular disease [3, 32]. mTOR is a negative regulator of autophagy, and its inhibitor rapamycin induces autophagy in most of cells [10, 33]. In this study, we found that METTL3 inhibited the level of p-mTOR, and facilitated autophagy activation. The mTOR activator MHY1485 can largely reverse METTL3-induced autophagy. During autophagy process, mTOR inactivation is crucial for autophagy initiation [34]. Indeed, we previous results showed that more autophagosomes formation was detected in METTL3 overexpressed VSMCs, and the expression of autophagy related 5 (ATG5) and ATG7 were increased by METTL3 overexpression [9]. Except for autophagy regulation, mTOR also contributes to proliferation regulation in an autophagy-dependent and -independent manner [13, 35]. For example, caffeine suppressed VSMC proliferation and induced autophagy by inhibiting mTOR signaling and WNT signaling in an in vivo vascular injury-induced restenosis model [1]. However, AdipoRon (a small-molecule adiponectin receptor agonist) can alleviate neointimal hyperplasia after angioplasty by targeting mTOR signaling independent of AMPK activation [13]. Rapamycin (Sirolimus)-eluting stent and rapamycin-coated balloon are widely used in clinical practice to treat coronary artery stenosis or prevent restenosis after stenting [36, 37]. We demonstrated that rapamycin promotes autophagy, migration, and synthetic phenotype of VSMCs, but inhibits VSMC proliferation. Given that we have demonstrated that the functions of METTL3 on VSMC proliferation, migration and phenotypic switching were autophagy-dependent [9], we believe mTOR is only partially involved in these functions of METTL3, and mTOR did not mediated the functions of METTL3 on migration and phenotypic switching of VSMCs.

CDK1 is the protein kinase that triggers S-G2 and G2-M transitions and G2 progression [38]. In our previous study, we found that METTL3 inhibited phosphorylation of CDK1 to arrest VSMC at G2 phase [9]. When we knockdown of CDK1 in HASMCs with METTL3 deficiency or not, CDK1 knockdown largely abrogated the pro-proliferation effects of METTL3 knockdown. Recently, CDK1 was found to be contribute to autophagy in mitosis and cell cycle, but it is still full of controversy. For example, Li et al*.* demonstrated that CDK1 promotes mitotic autophagy and cell cycle progression by inducing ULK1-ATG13 phosphorylation [39]. On the contrary, Odle et al*.* reported that autophagy initiation is repressed during mitosis, and CDK1 substitutes for mTORC1 to phosphorylate autophagy regulators such as ULK1, ATG13, ATG14, and TFEB [12]. In our study, we also found that in VSMCs with METTL3 knockdown, autophagy was repressed with CDK1 activation during mitosis. However, when we knockdown of CDK1 in VSMCs, mTOR was activated and the expression of ATG5, ATG7 and LC3II were downregulated, which indicated that autophagy initiation was suppressed by CDK1 deficiency. More importantly, CDK1 knockdown cannot counteract the role of METTL3 on autophagy in VSMCs. Thus, during mitosis of VSMCs, autophagy is regulated by mTOR but not CDK1, at least during METTL3 regulated proliferation of VSMCs.

## Conclusions

We found that METTL3 inhibits VSMC proliferation by activating autophagy, which is partially mTOR-dependent but CDK1-independent. These results not only provide evidences for the conditioned dependence of mTOR and CDK1 in regulating autophagy in mitosis, but also provide novel targets for the prevention and treatment of neointima formation.

## Methods and materials

### Materials

DME/F12 and fetal bovine serum (FBS) were purchased from Hyclone. 0.25% trypsin–EDTA was obtained from Gibco. The penicillin–streptomycin (PS) was from Geview. The primers of METTL3 were synthesized by Sangon Biotech (Shanghai, China), as well as Dimethyl sulfoxide (DMSO). MolPure Plasmid Mini Kit (19001ES70), Polyethylenimine Linear (PEI) MW40000 (rapid lysis) (40816ES02) and 5 × SDS Protein Loading (20315ES) were from Yeasen (Shanghai, China). MHY1485 (mTOR activator) and rapamycin (mTOR inhibitor) were purchased from Selleck. RIPA-lysis buffer (#HY-K1001) was purchased from MedChemExpress. Pierce™ BCA Protein Assay Kit (23227) were from Thermo Fisher Scientific. Polyvinylidene fluoride (PVDF) membranes (IPVH00010) were purchased from Millipore. The antibodies used in this study including anti-Flag (#H3663; 1:1000) obtained from Sigma; anti-β-actin (#AC026; 1:50000) obtained from Abclonal; anti-p-CDK1(#4539; 1:1000), anti-p-mTOR (#5536; 1:1000), anti-mTOR (#2983; 1:1000), anti-ATG5 (#12994; 1:1000), anti-ATG7 (#8558; 1:5000), anti-p-ULK1 (#5869; 1:1000) and anti-LC3 (#12741; 1:1000) obtained from Cell Signaling Technology; anti-PCNA (1:1000) obtained from Genetex; anti-p-Histone H3 (1:200) is from Santa Cruz; anti-METTL3 (15073-1-AP; 1:1000) and anti-CDK1 (10762-1-AP; 1:1000) obtained from Proteintech. Other antibodies such as anti-α-SMA (ab7817; 1:200), anti-SM22α (ab14106; 1:1000) are from Abcam. Secondary antibody (111–035-003) was purchased from Jackson ImmunoResearch Laboratories. Clarity Western ECL Substrate and Precision plus Protein Dual Color Standards were purchased from Bio-Rad Laboratories (USA). Transwell invasion chambers were obtained from Costar.

### Cell culture and treatments

Primary human aortic smooth muscle cells (HASMCs) were obtained from the ascending aorta when they underwent heart transplantation at Tongji Hospital, Tongji Medical College, Huazhong University of Science and Technology and cultured as described previously [20, 40, 41]. Simply, once the aortic wall tissues abandoned during donor trimming during heart transplantation were collected, they were quickly placed in pre-cooled DME/F12 medium and transferred to the laboratory in 4 °C. Subsequently, the intimal and residual adventitial tissues of the aortic wall were peeled under a stereomicroscope. Then, the media of the aortic tissue were cut into small pieces (1 × 1 mm) with the help of ophthalmic scissors. The clipped media of aortic tissues were cultured in DME/F12 medium supplemented with 10% fetal bovine serum and 1% antibiotics at 37 °C in a cell incubator containing 5% CO2. The medium was replaced after observation of long spindle-shaped smooth muscle cells around the tissue pieces. The density and status of the cells are closely monitored all the time and passed on to the next generation when the growth density reaches 80% until the third generation is used for experiments.

### Lentivirus production

The human plasmids of METTL3 and knockdown were previously described by us [9, 20, 42]. In brief, the full-length human METTL3 CDS sequence was amplified by polymerase chain reaction (PCR) and cloned into the pHAGE lentiviral vector with a Flag tag. The primers used to amplify the CDS of METTL3 were as follows: METTL3 forward primer: 5ʹ-CCGACGCGTGCCACCATGTCGGACACGTGGAG-3ʹ, METTL3 reverse primer: 5ʹ-ACGCGTCGACTAAATTCTTAGGTTTAGAGATGATAC-3’. Double-strand oligonucleotides of shRNA targeting human CDK1 were cloned into pLKO.1 plasmids. The target sequences of shCDK1 were: 5ʹ-GATTCAGAAATTGATCAACTC-3ʹ. For lentivirus production, 6 µg of plasmid DNA, 3 µg of psPAX2, 3 µg of pMD2.G, and 18 µL of PEI were mixed and added to HEK293T cells in a 10 cm culture dish. Medium was changed 8 h after transfection, and the supernatant was collected at 24 and 48 h posttransfection.

### Western blot analysis

Western blot was performed as previously reported [7, 10]. Briefly, the total protein from HASMCs with indicated treatment was extracted by RIPA-lysis buffer and quantified using Pierce™ BCA Protein Assay Kit. The protein was denatured at 95 ℃ with 5 × Loading buffer, 20 μg of total protein was loaded and separated by 10–12% SDS-PAGE, then was transferred to a PVDF membrane. Furtherly, after blocked by 5% skimmed milk for 1 h, the membrane was incubated with primary antibodies specific for METTL3, p-CDK1, CDK1, p-ULK1, LC3, PCNA, p-H3, ATG5, ATG7, α-SMA, SM22α, p-mTOR, mTOR and β-actin overnight at 4 °C. Subsequently, after primary antibody incubation, the membranes were washed in TBST and incubating with the secondary antibody (1:25000 dilution) at room temperature for 2 h. Then, the protein signals were observed and detected using the ChemiDocTM XRS + system (Bio-Rad). Proteins' expression was quantified using Image Lab.

### Transwell cell invasion assay

Transwell was used to detect the migration of HASMCs as previously reported [9, 11]. After 24 h of incubation with serum-free medium, cells were digested and resuspended in medium containing 0.5% FBS. Then, a total of 100 μL of cell suspension (2.5 × 10^4^ cells) was seeded onto the upper chambers of a transwell culture plate and 800 μL of medium containing 10% FBS was added to the bottom chamber after adherence for 3 h. The plate was incubated at 37 °C and 5% CO_2_ for 12 h. The cells in lower chamber were fixed with 4% paraformaldehyde fixative for 15 min and stained with 0.1% crystal violet for 30 min, washed away gently with water. Unmigrated cells on the upper chamber surface of the well were mildly wiped away by wet cotton swabs. Finally, the cells were observed and photographed with a microscope.

### Real-time PCR

Total mRNA was extracted with TRI Reagent^®^ Solution (A33251, Invitrogen), and 4 μg of total mRNA was reverse transcription into cDNA by using Hifair^®^ II 1st Strand cDNA Synthesis Kit (gDNA digester plus) (11119ES60, Yeasen). Then, PCR amplification was performed using Hifair^®^ qPCR SYBR^®^ Green Master Mix (No Rox) (11201ES08, Yeasen) in CFX connect™ real-time PCR detection system (Bio-Rad). The mRNA expression levels were normalized to 18S. Primers used in this study were as follow: ATG5 forward primer 5′-GCTTCGAGATGTGGTTTGG-3′ and ATG5 reverse primer 5′-CCATTTCAGTGGTGCCTTC-3′; ATG7 forward primer 5′-GCATCCAGAAGGGGGCTATG-3′ and ATG7 reverse primer 5′-GATCAAGAACCTGGTGAGGCA-3′; CDK1 forward primer 5′-CTGGGGTCAGCTCGTTACTC-3′and CDK1 reverse primer 5′-TCCACTTCTGGCCACACTTC-3′.

### Statistical analyses

The data were analyzed by GraphPad Prism 8 software in this study. All the data were expressed as mean ± standard deviation (SD). The differences between the two groups were determined using the student t-test, and statistical differences between multiple groups were analyzed using a one-way analysis of variance (ANOVA). A p value less than 0.05 is considered statistically significant.

### Supplementary Information

**Additional file 1: Figure S1.** The mRNA levels of ATG5 and ATG7 were upregulated in HASMCs with METTL3 overexpression. (A) Quantification of mRNA levels of ATG5 in HASMCs with or without METTL3 overexpression. (B) Quantification of mRNA levels of ATG7 in HASMCs with or without METTL3 overexpression. *p < 0.05 versus lenti-Flag. **Figure S2.** CDK1 was significantly knockdown in VSMCs. (A-B) The protein level of CDK1 was evaluated by using western blotting in HASMCs with CDK1 knockdown or not. (B) Quantitative results of blots in A (n = 4). (C) Quantification of mRNA levels of CDK1 in HASMCs with or without CDK1 knockdown. * p < 0.05 versus lenti-shRNA.

## Data Availability

Data are available from corresponding author upon reasonable request.

## Abbreviations

VSMC: Vascular smooth muscle cell
METTL3: Methyltransferase like 3
p-mTOR: Phosphorylated mammalian target of rapamycin
p-CDK1/CDC2: Cyclin dependent kinase 1
EZH2: Zeste homolog 2
EHMT2: Euchromatic histone lysine methyltransferase 2
DMSO: Dimethyl sulfoxide
HASMCs: Primary human aortic smooth muscle cells
