# Supplementary material for: The roles of METTL3 on autophagy and proliferation of vascular smooth muscle cells are mediated by mTOR rather than by CDK1
Source: Cell Div. 2023 Aug 9;18:13. doi: 10.1186/s13008-023-00096-5 (PMC10411010; doi:10.1186/s13008-023-00096-5)
Supplement: Supplementary file 1 — Additional file 1: Figure S1. The mRNA levels of ATG5 and ATG7 were upregulated in HASMCs with METTL3 overexpression. (A) Quantification of mRNA levels of ATG5 in HASMCs with or without METTL3 overexpression. (B) Quantification of mRNA levels of ATG7 in HASMCs with or without METTL3 overexpression. *p < 0.05 versus lenti-Flag. Figure S2. CDK1 was significantly knockdown in VSMCs. (A-B) The protein level of CDK1 was evaluated by using western blotting in HASMCs with CDK1 knockdown or not. (B) Quantitative results of blots in A (n = 4). (C) Quantification of mRNA levels of CDK1 in HASMCs with or without CDK1 knockdown. * p < 0.05 versus lenti-shRNA. [file 13008_2023_96_MOESM1_ESM.docx]

**Additional Figures**


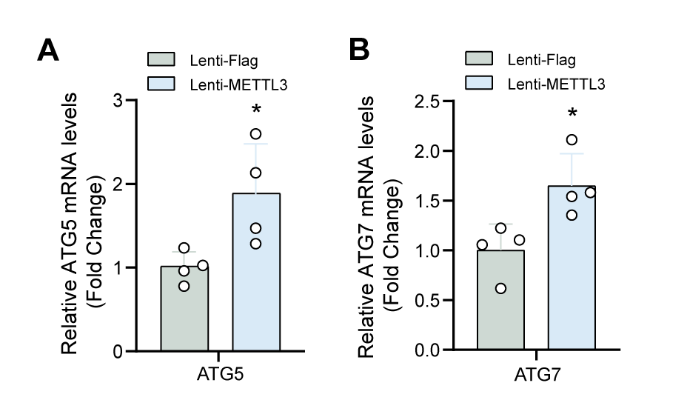


**Figure S1. The mRNA levels of ATG5 and ATG7 were upregulated in HASMCs with METTL3 overexpression. (A)** Quantification of mRNA levels of ATG5 in HASMCs with or without METTL3 overexpression. **(B)** Quantification of mRNA levels of ATG7 in HASMCs with or without METTL3 overexpression. *p < 0.05 versus lenti-Flag.


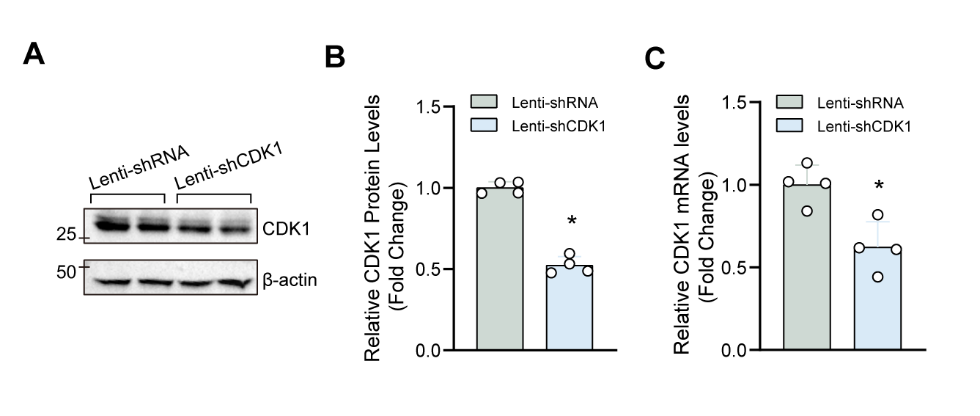


**Figure S2. CDK1 was significantly knockdown in VSMCs. (A-B)** The protein level of CDK1 was evaluated by using western blotting in HASMCs with CDK1 knockdown or not. (B) Quantitative results of blots in A (n = 4). **(C)** Quantification of mRNA levels of CDK1 in HASMCs with or without CDK1 knockdown. * p < 0.05 versus lenti-shRNA.
